# Developmental Pathway of the MPER-Directed HIV-1-Neutralizing Antibody 10E8

**DOI:** 10.1371/journal.pone.0157409

**Published:** 2016-06-14

**Authors:** Cinque Soto, Gilad Ofek, M. Gordon Joyce, Baoshan Zhang, Krisha McKee, Nancy S. Longo, Yongping Yang, Jinghe Huang, Robert Parks, Joshua Eudailey, Krissey E. Lloyd, S. Munir Alam, Barton F. Haynes, NISC Comparative Sequencing Program, James C. Mullikin, Mark Connors, John R. Mascola, Lawrence Shapiro, Peter D. Kwong

**Affiliations:** 1 Vaccine Research Center, National Institute of Allergy and Infectious Diseases, National Institutes of Health, Bethesda, Maryland, United States of America; 2 Laboratory of Immunoregulation, National Institute of Allergy and Infectious Diseases, National Institutes of Health, Bethesda, Maryland United States of America; 3 Duke Human Vaccine Institute, Duke University Medical Center, Durham, North Carolina United States of America; 4 NIH Intramural Sequencing Center, National Human Genome Research Institute, National Institutes of Health, Bethesda, Maryland United States of America; 5 Department of Biochemistry and Molecular Biophysics, Columbia University, New York, United States of America; Shanghai Medical College, Fudan University, CHINA

## Abstract

Antibody 10E8 targets the membrane-proximal external region (MPER) of HIV-1 gp41, neutralizes >97% of HIV-1 isolates, and lacks the auto-reactivity often associated with MPER-directed antibodies. The developmental pathway of 10E8 might therefore serve as a promising template for vaccine design, but samples from time-of-infection—often used to infer the B cell record—are unavailable. In this study, we used crystallography, next-generation sequencing (NGS), and functional assessments to infer the 10E8 developmental pathway from a single time point. Mutational analysis indicated somatic hypermutation of the 2^nd^-heavy chain-complementarity determining region (CDR H2) to be critical for neutralization, and structures of 10E8 variants with V-gene regions reverted to genomic origin for heavy-and-light chains or heavy chain-only showed structural differences >2 Å relative to mature 10E8 in the CDR H2 and H3. To understand these developmental changes, we used bioinformatic sieving, maximum likelihood, and parsimony analyses of immunoglobulin transcripts to identify 10E8-lineage members, to infer the 10E8-unmutated common ancestor (UCA), and to calculate 10E8-developmental intermediates. We were assisted in this analysis by the preservation of a critical D-gene segment, which was unmutated in most 10E8-lineage sequences. UCA and early intermediates weakly bound a 26-residue-MPER peptide, whereas HIV-1 neutralization and epitope recognition in liposomes were only observed with late intermediates. Antibody 10E8 thus develops from a UCA with weak MPER affinity and substantial differences in CDR H2 and H3 from the mature 10E8; only after extensive somatic hypermutation do 10E8-lineage members gain recognition in the context of membrane and HIV-1 neutralization.

## Introduction

Although antibodies against the human immunodeficiency virus type 1 (HIV-1) are slow to appear, after years of chronic infection, roughly half of HIV-1-infected donors develop antibodies capable of neutralizing half of circulating HIV-1 strains [1,2]. These antibodies provide potential templates to guide HIV-1 vaccine strategies [3,4], and details of their generation and maturation have generated intense interest. One means to delineate the developmental pathway of a particular antibody involves a) longitudinal sampling of donors from time of infection, b) next-generation sequencing of B cell transcripts, and c) identification of members of the antibody lineage in the longitudinal samples. Thus far, this has been accomplished for three antibody lineages: the CD4-binding-site-directed CH103 lineage from donor CH505 [5], a second CD4-binding site-directed lineage, CH235, from the same donor [6] and the V1V2-directed VRC26 lineage from donor CAP256 [7]. While details of the developmental pathway for each of these lineages have proven a boon to the HIV-vaccine field, few broadly neutralizing antibodies have been identified from longitudinal samples; most broadly neutralizing antibodies have been identified from cross-sectional samples, in which only a few (often one) time points are available. One such antibody, the 10E8 antibody from donor N152, is perhaps the broadest of all HIV-1 antibodies thus far identified [8].

The 10E8 antibody recognizes an epitope in the membrane-proximal external region (MPER) of the HIV-1 gp41 transmembrane glycoprotein. 10E8 belongs to a category of broadly neutralizing antibodies that includes antibodies 2F5 [9–11], 4E10 [12], and m66.6 [9,11,13,14]. Like other MPER-directed antibodies, the 10E8 antibody is able to recognize its gp41 epitope in the membrane context [10,15–17], a trait that it appears to acquire during maturation, as this trait is not present in V gene-reverted germline versions of these antibodies. Both membrane-context recognition and developmental uncertainty have generated considerable speculation about the origin of MPER-directed antibodies. Were they generated in response to other non-HIV-1 antigens [18]? Were the initial B cell recombinants polyreactive [19]? Or did the membrane-reactivity arise through somatic hypermutation (SHM) in the periphery, where autoreactivity checkpoints are less stringent [20]?

To address these questions, we attempted to delineate the developmental pathway of the 10E8 antibody. Unfortunately, PBMCs from donor N152, the source of antibody 10E8, were only sampled at two time points: January and June 2011, both more than 10 years after HIV-1 infection of donor N152; this sparseness of sampling complicated the identification of early lineage members. We previously used next-generation sequencing (NGS) of B cell transcripts from the January 2011 time point to identify 10E8-lineage member sequences in both heavy and light chains and to pair them phylogenetically [18]. Here we combined an additional round of NGS from the same January 2011 time point with structure-function studies of germline and mature 10E8 antibodies and of their maturation intermediates to approximate the developmental pathway for the 10E8 lineage. The results provide insight into the nature of the antigen that initially stimulated the 10E8 lineage, the extent of polyreactivity in the B cell recombinant, and the acquisition of the ability of 10E8 to recognize membrane-bound antigens.

## Materials and Methods

### Human specimen

Sera and peripheral blood mononuclear cells (PBMCs) were obtained from an HIV-1-infected donor (N152) enrolled in a clinical protocol approved by the Investigational Review Board in the National Institute of Allergy and Infectious Diseases (IRB-NIAID). An IRB-NIAID approved consent form was provided to all donors, which was reviewed with all donors by a NIAID clinician. Donor N152 provided written consent to use of these samples [18,21].

### Antibody expression and purification

Codon-optimized genes corresponding to the 10E8 heavy and light chain intermediates were synthesized with a 5’ secretion leader sequence and sub-cloned into the mammalian expression vector pVRC-8400. Transient expression of the antibodies and variants was undertaken in 293F cells or 293 Expi cells as per manufacturer’s instructions (Life Technologies) by co-transfection of equal amounts of the respective heavy and light chain plasmids. Antibody IgG’s were purified from expression supernatants by capture with Protein A sepharose (Pierce) followed by elution with low pH elution buffer (Pierce) and adjustment of eluate pH with use of Tris-Cl (pH 8.0).

### Antibody antigen binding fragments (Fabs)

The antigen binding fragments of 10E8 intermediates were prepared using endoproteinase Lys-C (Roche) digestion as previously described [18]. Briefly, the protease was added at concentrations of 1:1,000 to 1:10,000 and the digestion undertaken at 37°C for 4–6 h. Protease digestion reactions were stopped with protease inhibitor tablets (Roche) and cleaved products were passed over a Protein A column to remove the Fc fragment.

### Mutagenesis of 10E8 reverted gHv and gLv antibodies

10E8 gHv and gLv antibodies were prepared by using the assigned germline genes with the fully mature CDR H3 sequence as defined in Huang et al.,[21]. Initial tests with this Vgene-reverted antibody showed no neutralization against a set of HIV-1 pseudoviruses, while neutralization was observed with a substantially reverted antibody with no mutations in the framework regions of the antibody and with ten mutations in CDR H1 and CDR H2 and two mutations in the CDR L1[7]. Initial neutralization tests using a fully reverted gLv antibody combined with a heavy chain containing eight mutations in the CDR H2 and a fully mature CDR H3 (10E8 8H) gave reasonable potency against a panel of eight pseudoviruses. Further mutagenesis was carried out on this construct along with neutralization assays to identify a single K52T mutation as sufficient to enable neutralization of seven out of eight of these strains with reasonable potency. All mutagenesis experiments were performed by GeneImmune Biotechnology (Maryland, US) using QuickChange II Site-directed mutagenesis Kit (Agilent Technologies, Santa Clara, US). The respective mutations were confirmed by DNA sequencing. Antibody mutants were expressed by pairing with corresponding wild type partner chains following the same protocol described for wild type IgG expression. All antibodies were purified by affinity chromatography using Protein A columns and eluted by IgG Elution Buffer (Pierce). The antigen binding fragments (Fab) of all VRC01-class antibodies were generated by Lys-C (Roche) digestion of IgG as previously described [22].

### Crystallization, structure determination, and structural analysis

Antigen-binding fragments (Fabs) of 10E8 variants with V-gene regions reverted to genomic origin for heavy-chain, but with mature light chain (termed here gHv/mature L) or with V-gene regions reverted to genomic origin for heavy and light-chains (gHv/gLv), or with mature heavy and light chains (mature H and L) were prepared and screened against 576 crystallization conditions adapted from the commercially available Hampton (Hampton Research), Precipitant Synergy (Emerald Biosystems) [23], and Wizard (Emerald Biosystems) screens using a Cartesian Honeybee crystallization robot by the vapor diffusion method in sitting drops at 20°C. Initial crystal hits were manually reproduced in hanging drops by mixing 0.5 μl protein complex solution with 0.5 μl reservoir solution.

Crystals of ligand-free mature H and L 10E8 Fab were obtained in a condition comprised of 6% PEG 8000, 25% Isopropanol, 0.1 M Imidazole pH 6.5, and were flash frozen in liquid nitrogen in mother liquor. Crystals of the gHv/mature L Fab were obtained in a condition comprised of 21% PEG 8000, 0.17M ZnAc, MES, pH6.0 and were flash frozen in liquid nitrogen in mother liquor supplemented with 22% glycerol. Crystals of the gHv/gLv Fab were obtained in a condition comprised of 15% PEG 8000, 10% Isopropanol, imidazole, pH 6.5 and were flash frozen in liquid nitrogen in mother liquor supplemented with 15% ethylene glycol.

Diffraction data for all crystals were collected at a wavelength of 1.00 Å at beamlines SER CAT ID-22 or BM-22 (Advanced Photon Source, Argonne National Laboratory). All diffraction data were processed using HKL-2000 [24]. Structures were solved with molecular replacement using Phaser [25] and refinement of the structures was carried out using a cross validation (R_free_) test set consisting of 5% of the data, using Phenix [26] with iterative rounds of model building in Coot [27]. Structure validation was carried out using MolProbity [28].

The ligand-free 10E8 Fab structure was refined to a final R_free_ value of 21.54% with 97.0% of the residues in the favored region of the Ramachandran plot, and 0% outliers. The gHv/mature L Fab structure was refined to a final R_free_ value of 27.1% with 98.6% of the residues in the favored region of the Ramachandran plot, and 0% outliers. The gHv/gLv Fab structure was refined to a final R_free_ value of 20.9% with 97.9% residues in the favored region of the Ramachandran plot, and 0% outliers. All structure figures were prepared using Pymol [29].

### HIV-1 neutralization assays

Neutralization was measured using a panel of eight HIV-1 Env-pseudoviruses to infect TZM-bl cells as previously described [30] (see S1 Table for details on HIV-1 isolate panel). Neutralization curves were fit using nonlinear regression on a 5-parameter Hill equation. The data were calculated as a reduction in luminescence units compared with control wells, and reported as half-maximum inhibitory concentration (IC_50_) in micrograms per milliliter for monoclonal antibodies.

### Sample preparation for 454 pyrosequencing

Samples for 454 pyrosequencing were prepared using cDNAs from Donor N152 PBMCs generated in a previous study [18]. Briefly, mRNA was extracted from 33 million PBMCs (Oligotex kit, Qiagen), and reverse transcription was performed in multiple 35 μl-reactions, composed of 15 μl of mRNA, 3 μl of oligo(dT)_12-18_ at 0.5 μg/μl (invitrogen), 7 μl of 5x first strand buffer (Invitrogen), 3 μl of RNase Out (Invitrogen), 3 μl of 0.1M DTT (Invitrogen), 3 μl of dNTP mix (each at 10 mM), and 3 μl of SuperScript II (Invitrogen). Immunoglobulin gene-specific PCRs were undertaken using 10 μl of the cDNA as template (equivalent of transcripts from 10 million PBMCs), 5 μl HiFi Taq 10x reaction buffer (Invitrogen), 2 μl 50 mM MgSO_4_, 2 μl dNTP mix (each at 10 mM), 2 μl gene-specific 5’-primer (S2 Table) at 25 μM, 2 μl of each 3’-primer at 25 μM, 1 μl DNA polymerase, and water to a final volume of 50 μl. The primers each contained adaptor sequences (XLR-A or XLR-B) for subsequent 454 pyrosequencing. PCRs were initiated at 95°C for 30 sec, followed by 25 cycles of 95°C for 30 sec, 58°C for 30 sec, and 72°C for 1 min, followed by 72°C for 10 min. PCR products were gel purified (Qiagen) and phenol/chloroform extracted. 454 pyrosequencing, instrumentation specifics, and initial data processing and sequencing were carried out as previously described [18].

### Bioinformatics analysis of 454 pyrosequencing-determined antibodyomes

The bioinformatics pipeline developed in our previous studies [18] was used to process the 454 pyrosequencing data for antibodyome analysis. We have made some changes to the original pipeline described below. As before, the pipeline consists of the following five steps: (i) filter out reads with less than 300 nt; (ii) assignment of variable (V), diverse (D), and joining (J) gene families and alleles to each sequence using an in-house implementation of IgBlast [31] (sequences with E value > 10^−3^ for V or J gene assignment were rejected); (iii) template-based error correction scheme, where 454 homopolymer errors in V, D (heavy chain only), and J regions were detected and corrected based on sequence alignment to the respective germ-line sequence; (iv) comparison with a set of template antibody sequences at both nucleotide and amino acid levels using a global alignment module in ClustalW [32]; and (v) determination of the nucleotide and amino acid sequences comprising the third complementarity-determining region (CDR H3 or L3). A preliminary analysis of the processed data showed very few sequences with the IGHD3-3*01 germline assignment which occurs in the mature heavy chain 10E8 sequence. We thus submitted all processed sequences containing the same V-germline assignment as the heavy chain 10E8 sequence to the IMGT High-Vquest server (http://www.imgt.org/IMGTindex/IMGTHighV-QUEST.html) [33]. This additional processing resulted in a larger of pool of sequences with the IGHD3-3*01 germline gene assignment.

We used a series of bioinformatic sieves to enrich the pool of sequences containing the same lineage characteristics as the mature 10E8 sequence for downstream phylogenetic analysis. These sieves were applied to both the heavy and light chain processed NGS data sets and removed any sequences that did not have the same germline gene assignments as in the mature 10E8 sequence. The first sieve was applied to both heavy and light chain NGS data sets and removed all sequences that did not have the correct V germline assignment (IGHV3-15). A second sieve was applied only the heavy chain and removed all sequences that did not have the IGHD3-3*01 D-gene assignment. A third sieve was applied to both the heavy and light chain data sets and removed all sequences that did not have the correct J germline gene assignment. Once sieving was done using the germline gene assignments both, heavy and light chain data sets were subjected to a second round of sieving using sequence signatures appearing in the CDR3 sequence. The signature sequence for the heavy chain derives from the IGHD3-3*01 germline gene, YYDFWSGYYT, and appears in the mature 10E8 sequence. For the heavy chain data set, sequences were sieved using the following sequence signature: Y[AD]FW[SG]GY, where the “[]”indicates either of the two amino acids were present at that position. Thus any sequence that contained either the full signature or a partial signature was retained for further downstream processing. A similar sieve was applied to the germline filtered light chain data set. However, since the number of sequences for the germline-sieved light chain data set was large (149,325), we applied a CDR L3 length filter followed by the CDR L3 signature filter. The length filter removed any sequence with a CDR L3 length (IMGT) shorter than 9 amino acids or longer than 13 amino acids. The length sieve had little effect on reducing the total number of sequences but did remove sequences residing outside the large contours. Application of the CDR L3 signature sieve was effective at pulling out mature sequences only.

### Phylogenetic analysis and the determination of heavy and light chain intermediates

After sieving both the heavy and light chain NGS data sets, we generated maximum-likelihood (ML) trees for both the germline-filtered and the CDR signature derived data sets. Before tree construction, sequences were vetted and corrected for any sequencing errors which couldn’t be removed using our template-based correction algorithm.

For the heavy chain data set, we constructed two separate multiple sequence alignments (MSA) from both the germline-derived sequences (235 sequences) and CDR H3 signature-derived sequences (50 sequences). The alignments were then provided as input to construct a maximum-likelihood phylogenetic tree using dnaml in the PHYLIP [34] package v3.69 (http://evolution.genetics.washington.edu/phylip.html). The calculation was done with default parameters and the outgroup was set as the germline gene and the resulting tree visualized using Dendroscope [35]. Intermediate sequences were corrected at nucleotide positions containing low probability scores that introduced stop codons. Ambiguous nucleotide positions were estimated based on the more mature sequences along the evolutionary pathway.

For the light chain data set, the number of plausible sequences for the CDR L3-derived data set was large (149,301 sequences) and so we first partitioned the ensemble of sequences into germline divergence bins separated by 0.5% units. A sequence from each bin was then randomly selected and the total pool of sequences aligned and then provided as input for ML tree construction using dnaml. In the event that any sequence contained an error (i.e., failed to translate without a stop codon) that sequence was discarded and another sequence randomly selected in its place. For the CDR L3 signature-sieved data set (513 sequences), all sequences containing the SSRDKSGSR signature in the CDR L3 were binned in a similar fashion aligned and then used as input for dnaml.

To evaluate the fitness of each UCA sequence, we looked at a number of sequence properties that we felt would best characterize the most plausible maturation pathway. The properties included the number of intermediates in the ML tree, the total number of reversions between the UCA and mature 10E8 and the number of N-type additions at the junctions for both heavy and light chain UCA sequences (see S3 and S4 Tables). The junctional analysis was performed using the program JOINSOLVER [35] and is available through a web-interface (http://joinsolver.niaid.nih.gov).

### Surface-Plasmon Resonance (SPR) binding analysis

A Biacore T200 (GE Healthcare) was used to assess binding affinity of 10E8 maturation intermediates to a gp41 MPER peptide. A biotinylated peptide composed of residues 656–683 of the gp41 MPER (RRR-NEQELLELDKWASLWNWFDITNWLWYIR-RRK-biotin) was coupled to a biacore SA chip and 10E8 intermediate Fabs flowed over as analytes at two-fold serial concentrations that ranged from 15.6–500 nM (UCA), 3.9–500 nM (pI1), 15.6–125 nM (pI2, pI3, and 10E8 mature), and 31.25–500 nM (17b). Association and dissociation phases extended up to 5 min, at a flow rate of 30 μl/min.

### Liposomes

Liposomes were prepared as previously described [15] and reflected lipid ratios predicted to be present in the HIV-1 lipid bilayer. A 9:9:18:20:45 molar ratio of 1,2-dioleoyl-*sn*-glycero-3-phosphatidylcholine (DOPC), 1,2-dioleoyl-*sn*-glycero-3-[phospho-rac-(1-glycerol)] (DOPG), egg sphingomyelin (SM), 1,2-dioleoyl-*sn*-glycero-3-phosphatidylethanolamine (DOPE), and cholesterol (CHOL), respectively, was used. All lipids were purchased from Avanti Polar Lipids, Inc. MPER proteoliposomes were embedded with an MPER peptide spanning gp41 residues 659–683 as previously described [15], to a final concentration of 16 μM. The final concentration of phospholipid in all liposomes was 50 mM.

### Liposomal MPER competition ELISA

A biotinylated MPER peptide corresponding to residues 656–683 of gp41 MPER (RRR-NEQELLELDKWASLWNWFDITNWLWYIR-RRK-biotin) was captured at 4°C overnight on neutravidin-coated ELISA plates (Pierce) at a concentration of 0.1–0.2 μM. Plates were washed in phosphate-buffered saline (PBS) blocked for 1 hr at 25°C in PBS supplemented with 5% fetal bovine serum plus 2% dried milk. 100 μl of each respective antibody in blocking buffer was incubated alone or in presence of respective MPER competitors at 5 fold serial concentrations ranging from 1x10^-3^ to 16 μM for 1 hr at 25°C. These mixtures were added to the wells coated with MPER peptide and incubated for an additional hr at 25°C. The plates were then washed in PBS and 100 μl of mouse horseradish peroxidase (HRP)-conjugated anti-human Fc IgG added at 1:10,000 dilution in blocking buffer to all wells. After 1 hr of incubation at 25°C, plates were washed and a Bio-Rad HRP substrate detection kit used to detect bound secondary antibody. The reactions were stopped by adding 0.5 N sulfuric acid, and optical densities were measured at 450 nm. In each case, experiments were run in duplicate.

### Antibody autoreactivity assays

Indirect immunofluorescence binding of rMAbs to HEp-2 cells (Inverness Medical Professional Diagnostics, Princeton, NJ) was performed as described previously (16). Briefly, 20 μl of antibody at 25 μg/ml was placed on a predetermined spot on the surface of an ANA HEp-2 kit slide, incubated for 25 min at room temperature, washed, and developed with 20 μl of goat anti-human Ig-FITC at 20 μg/ml (Southern Biotech, Birmingham, AL) for 25 min. For images of murine MAb 13D5, goat anti-mouse Ig-FITC at 20 μg/ml (Jackson ImmunoResearch, West Grove, PA) was used. Incubations were performed in humid chambers in the dark. Slides were washed and dried; a drop of 33% glycerol was placed on each spot prior to the fixing of coverslips. Images were taken on an Olympus AX70 with SpotFlex FX1520 charge-coupled device (CCD) with a UPlanFL 40x (numerical aperture, 0.75) objective at 25°C in the FITC channel using SPOT software. All images were acquired for the time specified in the figure legend. Image layout and scaling were performed in Adobe Photoshop without image manipulation. Monoclonal antibody reactivity to SSA/Ro, SS-B/La, Sm, ribonucleoprotein (RNP), Jo-1, double stranded DNA, centromere B, and histone was measured by using the AtheNA Multi-Lyte ANA kit (Alere Inc., Waltham, MA) as previously described [19]. Antibodies were incubated with AtheNA beads for 30min at final concentrations of 50, 25, 12.5 and 6.25 μg/ml. Beads were washed, incubated with secondary and read on the Luminex platform. Samples were analyzed using AtheNA software. Cardiolipin reactivity was measured using the Quanta Lite ACA IgG III kit (Inova Diagnostics, Inc. San Diego, CA) by diluting antibodies to final concentrations of 50, 25, 12.5 and 6.25 μg/ml in sample diluent and assaying per manufacturer’s specifications.

### Data

10E8 crystal structures determined here have been deposited with the Protein Data Bank (PDB) under PDB codes (5JNY, 5JO5, 5JR1). Functionally characterized sequences of antibody intermediates have been deposited with Genbank under accession codes KX147288- KX147295. NGS data from donor N152 obtained with the modified heavy chain primers have been deposited with the Short Reads Archives (SRA) under SRP018335.

## Results

### Structures of 10E8 variants with V_H_ regions reverted to germline

To gain insight into the structural changes that accompanied maturation of the 10E8 antibody from B cell recombinant to mature antibody, we determined crystal structures of the ligand-free antigen-binding fragments (Fabs) of the mature 10E8 antibody and two germline variants: (i) a V-gene reverted version (gHv/gLv), in which both the heavy chain and light chain variable regions were reverted to their germline origin, and (ii) a heavy chain-only version (gHv/mature L), in which only the heavy chain-variable region was reverted to its germline origin and paired with the mature 10E8 light chain. X-ray diffraction data from these three structures extended to 3.05, 1.70, and 1.60 Å respectively, and they were solved by molecular replacement and refined to R_work_/R_free_ of 18.8/21.5, 23.7/27.1, and 19.4/20.9% for ligand-free mature H and L, gHv/gLv, and gHv/mature L versions of 10E8, respectively (Table 1). Electron density for an unknown ligand was observed adjacent to residue Ser 100c in the CDR H3 for the gHv/mature L 10E8 Fab.

**Table 1:** Crystallographic data collection and refinement statistics.

	10E8 mature H and L	10E8 gHv/gLv	10E8 gHv/mature L
**PDB accession code**	5JNY	5JO5	5JR1
**Data collection**			
Space group	*P*6_3_	P2_1_	P2_1_2_1_2_1_
Cell constants			
*a*, *b*, *c* (Å)	248.6, 248.6, 54.3	115.3, 65.3, 128.1	68.8, 77.9, 85.0
α, β, γ (°)	90.0,90.0,90.0	90.0,90.0,90.0	90.0,90.0,90.0
Wavelength (Å)	1.00	1.00	1.00
Resolution (Å)	50.0–3.04 (3.16–3.05)*	50.0–1.50 (1.62–1.55; 1.55–1.50)	50.0–1.60 (1.72–1.66;1.66–1.60)
*R*_merge_ (%)	22.1 (68.4)	12.7 (54.4; 68.7)	13.0 (90.2; 95.2)
*I* / σ*I*	10.4 (2.09)	7.87 (1.63; 1.13)	9.83 (2.14; 1.38)
Completeness (%)	99.7 (98.3)	100.0 (100.0; 100.0)	84.6 (89.2;83.4)
Redundancy	10.6 (7.6)	4.0 (3.9; 3.8)	4.7 (4.3; 3.4)
**Refinement**			
Resolution (Å)	32.8–3.05 (3.16–3.05)	38.47–1.7 (1.76–1.70)	37.3–1.6 (1.63–1.60)
No. reflections	37411 (3610)	204611 (6502)	51383 (2443)
*R*_work_ / *R*_free_ (%)	18.76/21.54	23.7/27.1	19.4/20.9
No. atoms			
Protein	6727	13077	3323
Ligand/ion	25	0	5
Water	16	1700	308
*B*-factors (Å^2^)			
Protein	56.2	25.57	31.64
Ligand/ion	49.6	n/a	32.00
Water	38.0	32.3	37.18
**R.m.s. deviations**			
Bond lengths (Å)	0.003	0.005	0.012
Bond angles (°)	0.720	0.774	1.232
**Ramachandran**			
Favored (%)	97.00	98.54	97.9
Allowed (%)	3.00	1.46	2.08
Disallowed (%)	0.0	0.0	0.0

*Values in parentheses are for highest-resolution shell(s).

**Fig 1:**
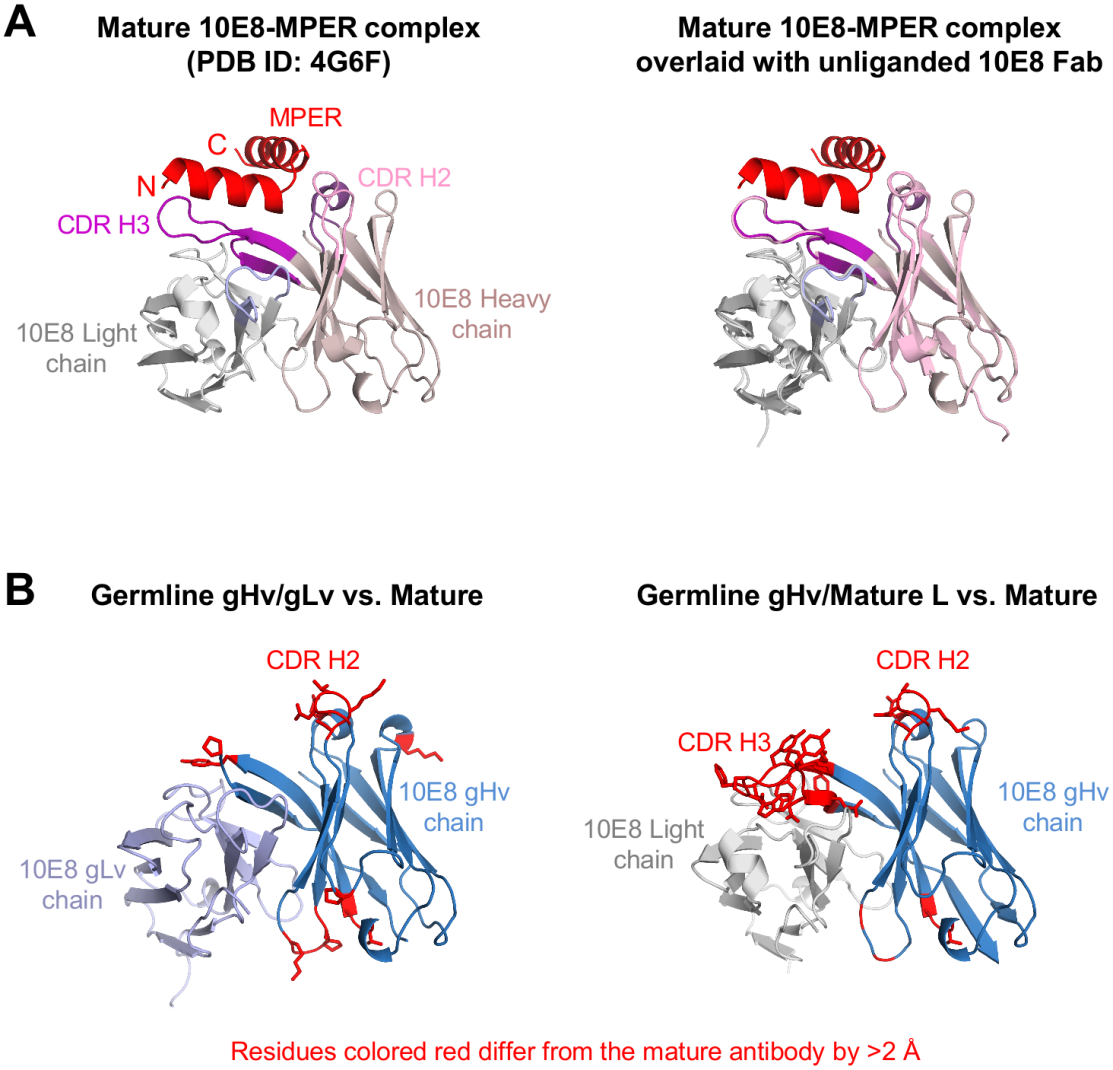
Structures of mature10E8 antibody and its genomic revertants. (**A**) (*left*) Mature antibody-MPER complex (PDB ID: 4G6F). The MPER peptide is colored red and the mature 10E8 antibody heavy chain in pink and light chain in gray. (*right*) The ligand-free 10E8 Fab is structurally aligned to the 10E8-MPER complex structure with an overall Cα r.m.s.d of 0.2 Å. (**B**) (*left*) Ligand-free structures of germline-reverted gHv/gLv and (*right*) partially reverted gHv/mature L structurally aligned to the mature 10E8 with an overall Cα r.m.s.d of 0.2 Å. Structural differences with Cα r.m.s.d. greater than 2.0 Å are colored in red and displayed in stick representation.

Comparison of the structure of the mature uncomplexed Fab with that of the previously determined structure of mature 10E8 bound to its gp41 epitope [8] showed minimal changes in the complementarity-determining regions (CDRs), with root-mean-square deviation (rmsd) for the entire Fv domain of 0.2 Å (Fig 1A). By contrast, the structure of the gHv/gLv Fab showed substantial changes in both the heavy chain 2^nd^ and 3^rd^ CDRs (CDR H2 and H3). All four residues at the top of the gHv/gLv CDR H2 differed in Cα-positions by over 1 Å from the mature unbound structure, and much of the CDR H3 was disordered (Fig 1B and S1 Fig). Differences were also seen in the gHv/mature L structure, though in this case, the CDR H3 was fully ordered. Thus maturation from germline to mature 10E8 affects the structure of the CDR H2 and H3 regions, and these structural changes occur primarily as a result of somatic hypermutation of the heavy chain V gene.

### Mutational analysis of CDR H2

**Fig 2:**
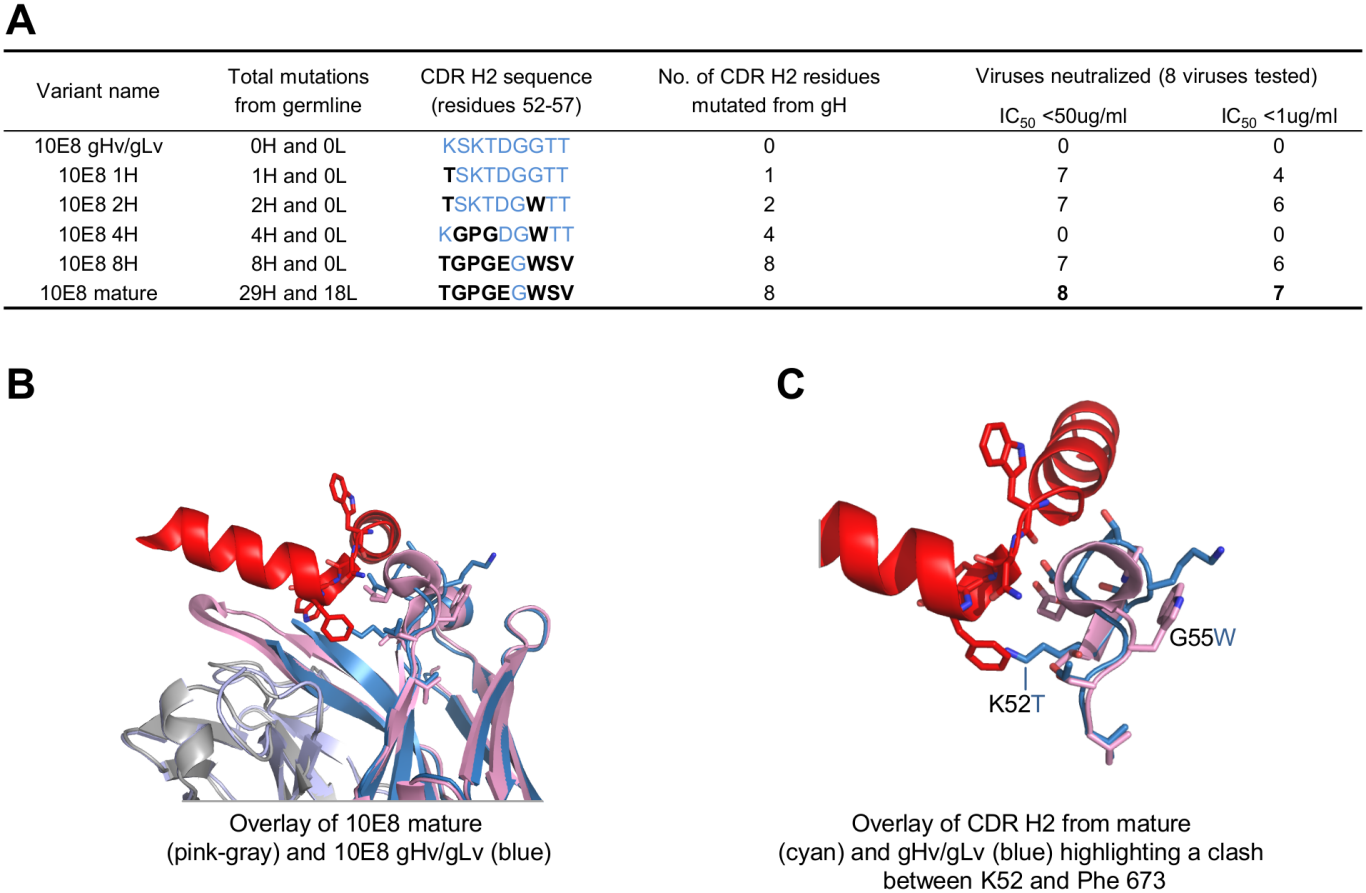
Analysis of 10E8 somatic hypermutation identifies critical K52T mutation. (**A**) Summary of CDR H2 somatic hypermutation and 10E8 neutralization. Total mutations from germline are indicated for both heavy and light chains. The CDR H2 germline sequence is shown colored blue with somatic mutations indicated in black. Viruses neutralized with an IC_50_ < 50 μg/ml or < 1 μg/ml are indicated (see S1 Table for details on HIV-1 isolate panel). (**B**) Superposition of mature 10E8 complex structure and 10E8 gHv/gLv. Coloring is the same as in Fig 1. (**C**) Expanded view of CDR H2. Residues that were mutated and gave improved 10E8 gHv/gLv neutralization levels are shown in stick representation. Residues Phe 673 (MPER) and Lys 52 (10E8 gHv/gLv) overlap and are not compatible with binding due to steric clashing.

To delineate the functional importance of specific residues mutated during V_H_-gene maturation, we analyzed sequence differences between mature and germline sequences. A cluster of eight out of nine residues were altered by SHM in the CDR H2 (Fig 2A). Reversion of the 29 and 18 mutations in the heavy and light V-gene regions, respectively, abolished neutralization (Fig 2A, 10E8 gHv/gLv), whereas retaining only the eight SHM-derived mutations in the CDR H2 retained neutralization for seven of eight diverse HIV-1 strains (Fig 2A, 10E8 8H). A single K52T mutation in CDR H2 allowed the otherwise V gene-reverted 10E8 to neutralize seven of eight strains (Fig 2A, 10E8 1H); while including a second mutation G55W increased potency to near wild-type levels (Fig 2A, 10E8 2H). (Antibody numbering is provided in Kabat nomenclature [36].)

Analysis of residue K52 indicated a clash with the MPER epitope at the highly conserved Phe 673 residue (Fig 2B); W55, in contrast, appears to stabilize the CDR H2 by forming hydrogen bonding interactions with residue 52b (Lys in germline and Pro in mature antibody; the “b” in 52b refers to the 2^nd^ insertion after residue 52 in Kabat nomenclature) allowing residue 53 (Asp or Glu) to hydrogen bond with the main chain N of Trp 672 of the MPER. Thus, critical SHM alterations for MPER recognition appear to be located in CDR H2 with the K52T mutation mediating the most significant change in recognition.

### Next-generation sequencing of B cell transcripts from donor N152

To identify 10E8 lineage members, we previously performed NGS of B cell transcripts from donor N152 [8], the source of the broadly neutralizing antibody 10E8, using polymerase chain reaction (PCR) to amplify IgG-heavy chain sequences from the IGHV3 family and to amplify IgG-light chain sequences from the IGLV3 family [18]. Because the 10E8 lineage heavy chain transcripts were not highly prevalent [18] and as a control for the error-prone nature of NGS, we performed a second set of PCR reactions for the 10E8 heavy chain to identify additional lineage members and to provide replicate data for error analysis (see methods and S2 Fig).

454 pyrosequencing was used to sequence the PCR products that were generated by an identical set of primers as the initial reactions from our previous study [18] with the exception of the 5’ heavy chain primer, XLR_A_VH3-15, which was modified to better correspond to VH3-15 antibody gene sequences (S2 Table). NGS generated a total of 647,551 reads of which 91% had sequence lengths greater than 300 nucleotides (S2 Fig). In our previous study [18], we obtained 843,084 heavy chain reads of which 98% had sequence lengths greater than 300 nucleotides. A comparison between the new and published NGS heavy chain data sets revealed the new data set to have a broader distribution of germline gene assignments.

### Bioinformatics identification of 10E8 lineage heavy-chain transcripts

**Fig 3:**
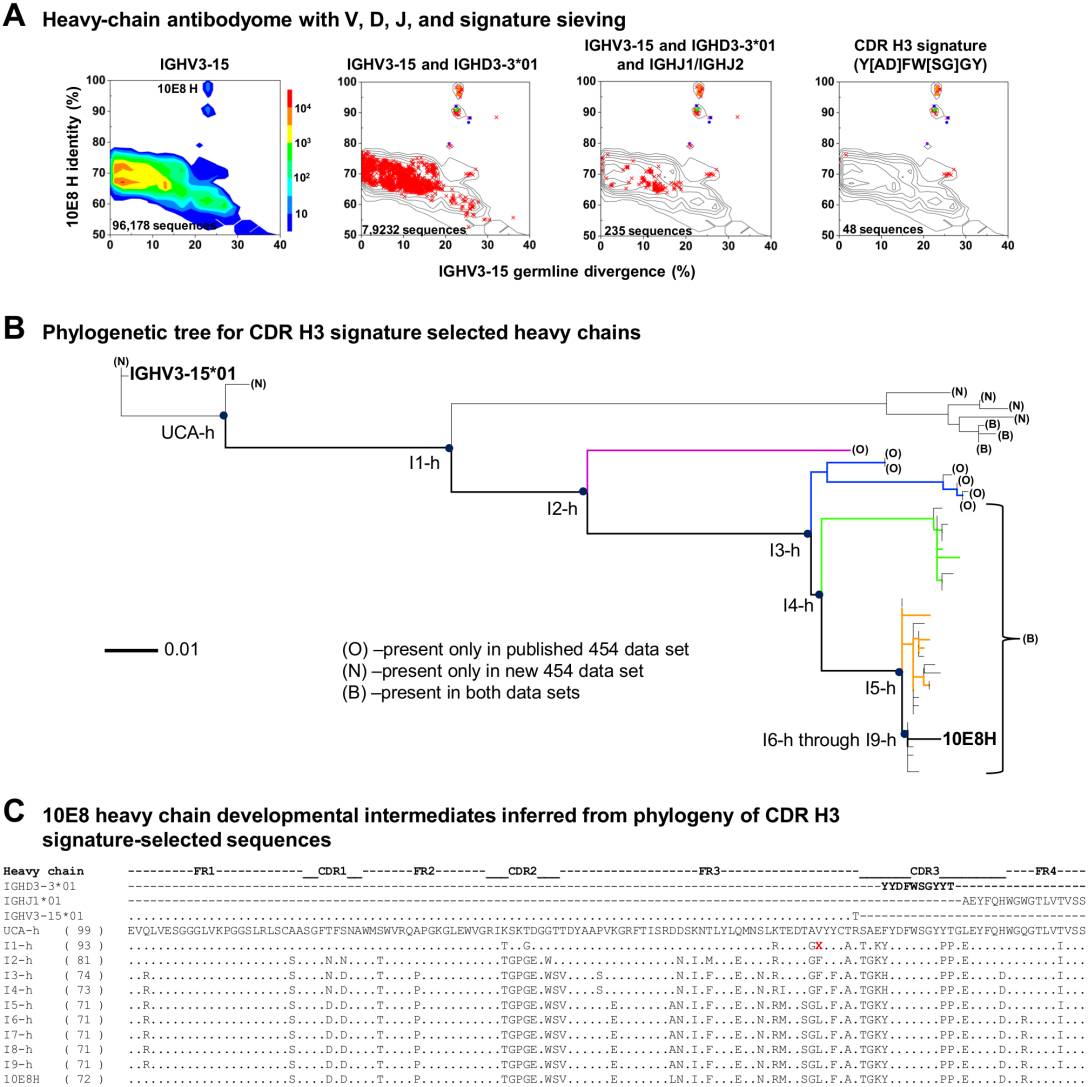
10E8 heavy chain ontogeny: lineage members and calculated intermediates. (**A**) Selection of 10E8 clonal variants by computational sieving. Identity/divergence plots showing the results of sieving on V gene (left), V, and J genes (panel 2), V, D, and J genes (panel 3), and CDR H3 signature (panel 4). (**B**) Phylogenetic tree based on CDR H3 signature-selected sequences; signature represents the overlap between the germline gene (HD3-3*01) and the mature 10E8 heavy chain sequence. Terminal branches were labeled to indicate which dataset the terminating sequence derives from. Branches ending ‘O’ indicate the sequence is derived from the published data set. Branches ending in ‘N’ indicate the sequence is derived from the new data set. And branches ending in ‘B’ indicate that the sequence can be found in both data sets. The threshold for inclusion into a data set was set at >97.5% nucleotide sequence identity and >98% alignment coverage. The coloring scheme for the ML tree was adapted from Zhu et al., [18]. Blue circles indicate nodal points on the tree. (**C**) Sequences of phylogenetically inferred developmental intermediates compared to mature 10E8 and constituent germline genes. Numbers appearing in parenthesis on the left side of the alignment indicate the number of identical residues to the germline V gene. The undefined residue labeled ‘X’ in I1-h was changed to a phenylalanine for pairing and functional analysis.

A total 96,178 full-length heavy chain sequences were assigned to IGHV3-15, of which 7,923 were derived from IGHD3-3, and 235 additionally derived from IgHJ1 and IgHJ2 (Fig 3A). Inspection revealed that a substantial number of these sequences contained a Y[AD]FW[SG]GY motif (bracketed pairs of amino acids signify a single position at which either amino acid appeared) in the CDR H3 region, which comprised an essential part of the 10E8 paratope and was remarkably similar to the 01 allele of IGHD3-3 (YYDFWSGYT). We therefore used the presence of this motif in the CDR H3 region to identify potential heavy chain-lineage members (Fig 3A, right most panel).

We constructed maximum-likelihood (ML) phylogenetic trees based on the CDR H3 signature-selected sequences (H3 signature tree) (Fig 3B), as well as on all sequences with the expected 10E8 V, D, and J origin genes (VDJ tree) (S3 Fig). While the VDJ tree defined over 30 intermediates between the potential UCA and mature 10E8, the UCA derived from this tree required 27 N-nucleotide additions and 64 reversions where each reversion comprises a nucleotide that mutates by SHM, but then reverts back to the original nucleotide during the predicted maturation pathway (S3 Table). In contrast, the H3 signature tree defined over 9 intermediates and the derived UCA required 20 N-nucleotide additions and 10 reversions (Fig 3C and S3 Table). Parsimony analysis therefore suggests the H3 signature tree to provide a more likely representation of 10E8 heavy chain development. Intermediates I1-h through I9-h, corresponding to each of the nodal points in the phylogenetic tree separating UCA from mature 10E8, were estimated and all nucleotide positions for each intermediate could be uniquely defined with the exception of a single position in the framework-3 region of intermediate I1-h (Fig 3C).

Although the additional set of 454 data did provide more sequences, as shown in the H3 signature tree, both data sets combined provide few low-divergence heavy chain reads making the determination of the UCA and I1-h intermediates imprecise (Fig 3B). In total, 73% of the heavy chain reads in the H3 signature tree appeared in both sets with the remaining sequences being split between the two sets. While the new NGS data provided a few more low-divergence reads than the published data set [18], none of the new low-divergence reads were duplicates. Sequences that appeared in both data sets clustered in the lower half of the ML tree (Fig 3B).

### Bioinformatics identification of 10E8 lineage light-chain transcripts

**Fig 4:**
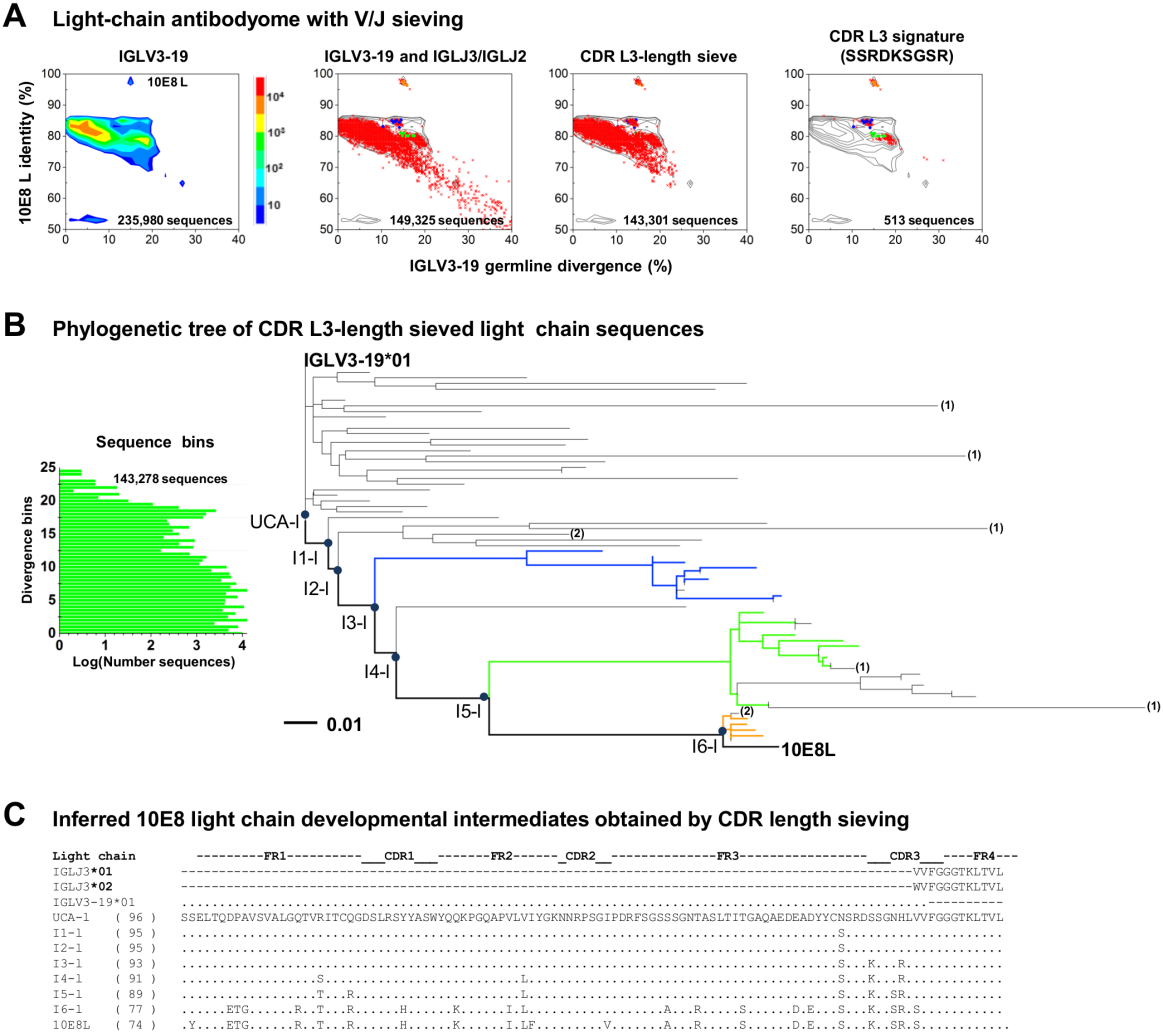
10E8 light chain ontogeny: lineage members and calculated intermediates. (**A**) Selection of 10E8 clonal variants by computational sieving. Identity/divergence plots showing the results of sieving on V gene (left), V, and J genes (panel 2), CDR L3 length between 9 and 12 residues (IMGT numbering) (panel 3), and CDR L3 signature (panel 4). (**B**) Phylogenetic tree based on CDR L3 length-selected sequences. To construct a phylogenetic tree from >10^5^ sequences, representative sequences were selected randomly from bins of 0.5 divergence units (as shown in left inset). The numbers in parenthesis at the end of each branch correspond to the total number of clones that share >99% nucleotide sequence identity and 99% alignment coverage. Branches terminating without numbers indicate that the sequence has more than 10 clones with >99% nucleotide sequence identity and 99% alignment coverage. The coloring scheme for the ML tree was adapted from Zhu et al., [18]. Blue circles indicate nodal points on the tree. (**C**) Sequences of phylogeneticaly inferred developmental light chain intermediates compared to mature 10E8 and constituent germline genes. Numbers appearing in parenthesis on the left side of the alignment indicate the number of identical residues to the germline V gene.

A total of 235,980 full-length light chain sequences were assigned to IGLV3-19, of which 149,325 were derived from IGLJ3 and IGLJ2. As we did not expect large insertions or deletions, we additionally analyzed sequences with CDR L3 lengths from 9 to 13 amino acids (IMGT nomenclature)–reducing the total number of sequences to 143,301 (Fig 4A). Inspection of the light chain sequence of mature 10E8 as well as the light chain sequences we previously identified to be part of the lineage [18] revealed all contain a SSRDKSGSR sequence motif in the CDR L3 region. We therefore used the presence of this 9-mer sequence as a sieve to identify potential light chain-lineage members (Fig 4A, right most panel).

We used the sieved light chain sequences to construct two ML phylogenetic trees: one based on the CDR L3 signature-selected sequences (L3 signature tree) (S4 Fig), and one based on the length-sieved sequences with expected 10E8 V and J origin genes (VJ tree) (Fig 4B). As there were too many length-sieved sequences to analyze by maximum likelihood, we sorted these sequences into 0.5% SHM divergence bins, and constructed a phylogenetic tree from randomly selected binned sequences. The L3 signature and the VJ tree produced different overall topologies with the former having only three nodal points (labeled as I1-l through I3-l) between the UCA and the mature 10E8 light chain sequence (S4 Fig). In contrast, the VJ tree has twice as many nodal points between the calculated UCA and the mature 10E8 light chain sequence (Fig 4B). While both the VJ tree and L3 signature tree had almost no nucleotide reversions, the calculated UCA from the L3 signature tree had 14 N-nucleotide additions in comparison to the UCA belonging to the VJ tree which had only 1 N-nucleotide addition (S4 Table). While the L3 signature tree was slightly more parsimonious than the VJ tree it lacked a well-defined UCA with 4 amino acid differences when compared to the IGLV3-19 germline gene (Fig 4C and S4 Fig).

### Pairing of heavy and light chain intermediates

NGS of antibodies that utilize separate heavy and light PCR reactions removes heavy-light pairing information. However, we previously showed that phylogenetic analyses could be used to pair antibodies of the 10E8 lineage, and that such phylogenetically paired antibodies exhibit reduced polyreactivity [18]. Based on our prior work, we attempted to use the topological information derived from the ML trees to obtain an approximate pairing for the heavy and light chain intermediates between the H3 signature tree and the VJ tree.

**Fig 5:**
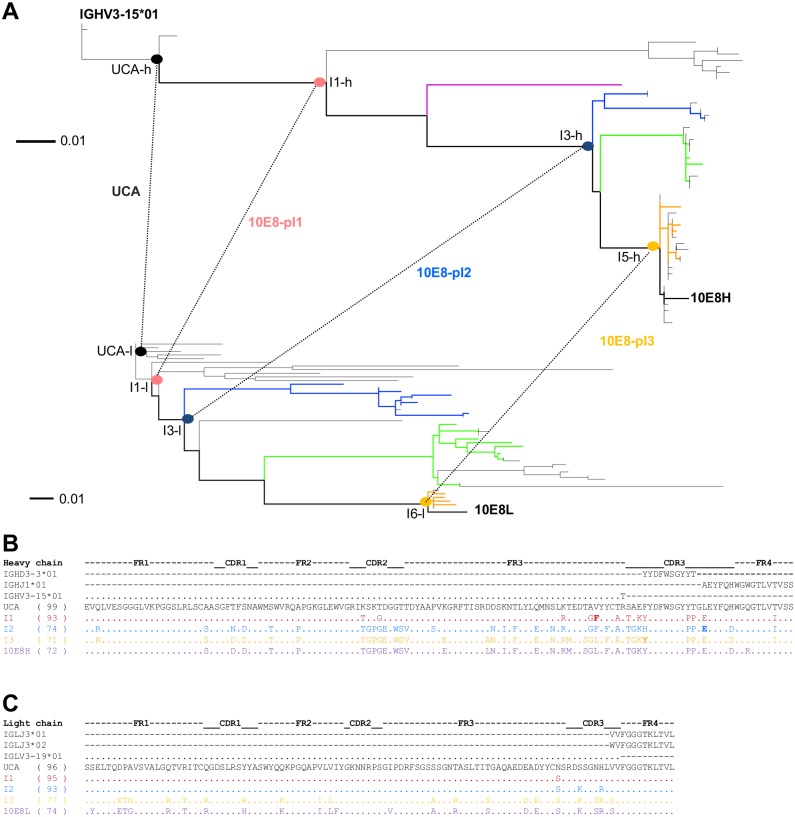
Pairing of heavy and light 10E8 intermediates. (**A**) Heavy (top) and light (bottom) phylogenetic trees with pairing of inferred intermediates indicated by dashed lines. Intermediate pairing based on tree structure and considerations from polyreactivity (see text and [18]). (**B**) Heavy chain UCA, intermediates and mature sequences. (**C**) Light chain UCA, intermediates and mature sequences.

The UCA and mature 10E8 define two unambiguous pairs. We paired the first intermediate sequence from each phylogenetic tree to create paired intermediate pI1 (10E8-pI1), as the early development of the lineage is of interest and I1 was the closest intermediate to UCA (Fig 5). Intermediate I2 left a number of possible nodal pairings between it and mature 10E8. One possibility would be to pair comprehensively all identified transcripts; however, as such pairing is only an approximation of 10E8 development, we decided that for a first assessment, we would approximate 10E8 development with only two additional pairings: pI2 (10E8-pI2, pairing I3-h and I3-l) and pI3 (10E8-pI3, pairing I5-h and I6-l) (Fig 5). The pairings were chosen to maintain the phylogenetic structure that previously displayed reduced polyreactivity [18].

### Functional assessment of 10E8 and maturation intermediates

**Fig 6:**
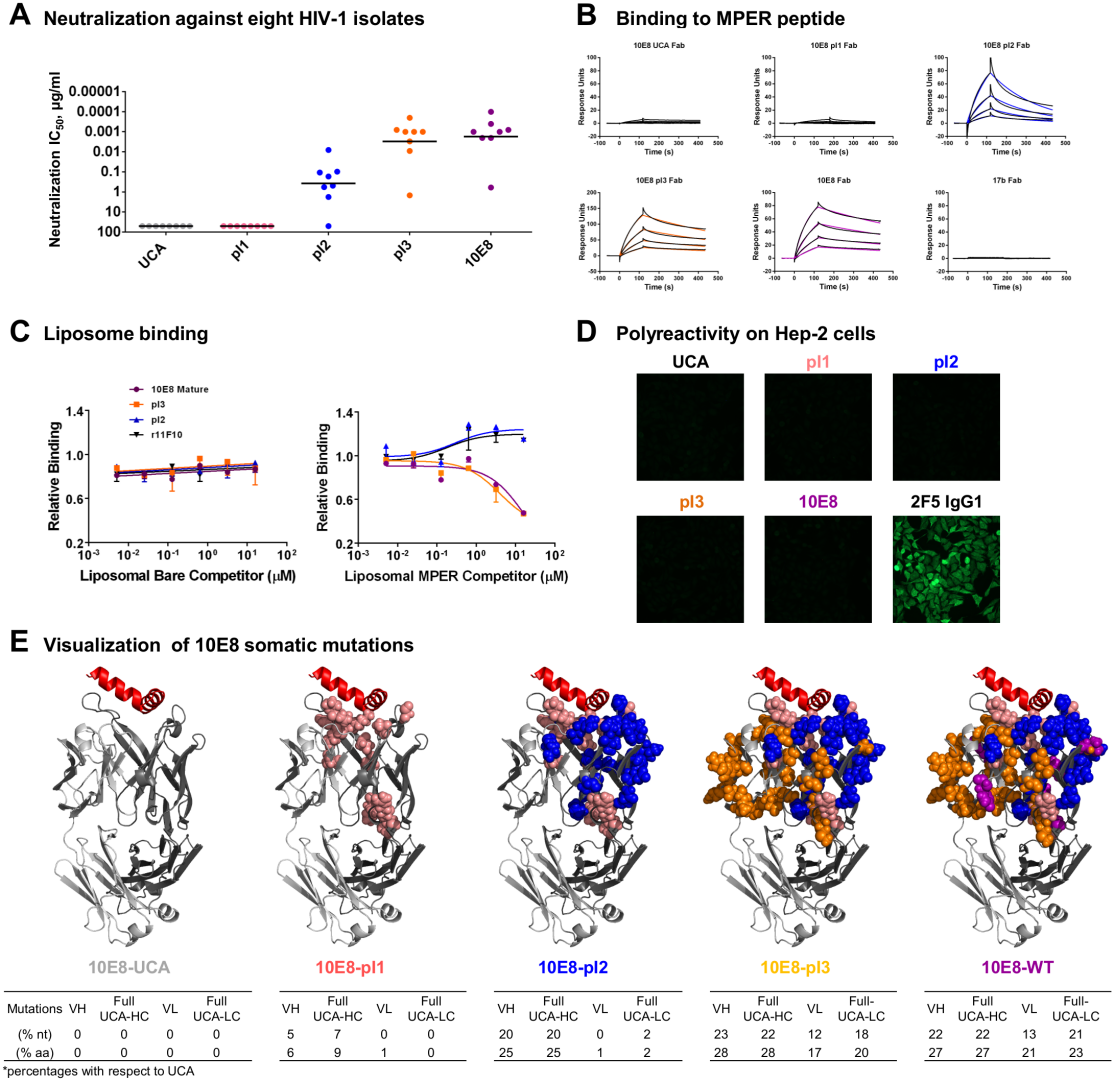
Functional characteristics of 10E8 UCA and maturation intermediates. (**A**) Neutralization on eight diverse HIV-1 isolates (see S6 Table for details on HIV-1 isolate panel). (**B**) Sensogram profiles shown represent two-fold serial dilutions of Fab analyte starting at top concentrations of 500 nM for UCA, pI1, and 17b, or 125 nM for pI2, pI3 and 10E8 mature, through to final concentrations of 3.9–31.25 nM. (**C**) ELISA assessment of MPER-liposome recognition. Shown are bare liposome (left) and MPER proteoliposome (right) competiton ELISA assays for antibody recognition of a soluble MPER peptide captured on a plate. Binding of 10E8 mature and pI3 antibodies to soluble MPER was effectively competed by MPER proteoliposomes in this assay. (**D**) Hep2 cell assessment of autoreactivity. (**E**) Structural models of 10E8 showing location and degree of somatic hypermutation by intermediate. Structural models of 10E8 intermediate antibodies shown in ribbon representation showing the location of resides mutated from the UCA as spheres and colored according to paired intermediate. The degree of somatic mutation for each 10E8 intermediate is given below each structure model with % nucleotide and % amino acid change from the germline VH gene and also for the calculated UCA gene.

The UCA, three 10E8 pairings (pI1, pI2 and pI3), along with mature 10E8 were expressed, purified and assessed for function. When tested for neutralization on eight diverse HIV-1 isolates, no neutralization was observed for UCA or pI1, while pI2 neutralized 7 or 8 strains, and pI3 and mature 10E8 showed increased potency against all 8 strains (Fig 6A). To assess functionality of the 10E8 maturation intermediates, fragments of antigen binding (Fabs) of each of the intermediates were produced and then tested in surface plasmon resonance (SPR) for binding to a peptide corresponding to residues 656–683 of the gp41 MPER (Fig 6B). Kinetic binding analysis revealed that the UCA, pI1 through pI3, and mature 10E8 bound gp41 MPER to varying levels. Both UCA and pI1 showed weak binding to the MPER peptide, although the signal observed was greater than that observed for a non-cognate control antibody 17b, whereas pI2 through mature 10E8 showed strong binding with decreasing off-rates (Fig 6B).

As functional recognition of the MPER appears to require binding in a lipid context [15], we also tested recognition of 10E8 variants to MPER utilizing an ELISA-based liposome competition assay. A biotinylated MPER peptide corresponding to residues 656–683 of gp41 was captured on neutravidin coated ELISA plates, and binding of the 10E8 maturation intermediates to captured peptide was assessed directly and then in the competitive presence of MPER-embedded proteoliposomes or bare liposomes. While binding of 10E8 UCA and pI1 to captured MPER peptide was too weak to detect in the direct binding assay (S5 Fig), and thus their corresponding MPER-lipid binding characteristics also could not be assessed, 10E8 mature, pI2 and pI3, all showed detectable binding to the captured soluble peptide, as did non-neutralizing MPER-specific antibody 11F10 that was used as a negative control [37] (S5 Fig). Addition of MPER-embedded proteoliposomes as a competitor revealed that only the 10E8 mature and pI3 antibodies could be effectively competed, while the binding of pI2 and 11F10 could not (Fig 6C). None of the antibodies were successfully competed for binding to soluble peptide by bare liposomes (Fig 6C). These results suggest that the capacity of paired 10E8 intermediates to recognize MPER in a lipid-context is a trait that appears to have arisen later in the maturation pathway, from pI3 and beyond.

We next addressed whether paired 10E8 intermediates were reactive with self-antigens. While mature antibody 10E8 has previously been shown to lack such auto-reactivity [8,18], we sought to determine whether this trait also applied to the paired intermediates. HEp-2 epithelial cell staining (Fig 6D) combined with examination of reactivity with a panel of anti-nuclear antigens (ANA) (S6 Fig) revealed an overall absence of reactivity with self-antigens, while control antibodies 2F5 and 4E10 exhibited high degrees of autoreactivity (Fig 6D, S6 Fig, S5 Table). These results suggest that 10E8 and its ancestral maturation intermediates lack reactivity with self-antigens from inferred inception, despite the capacity in some cases to recognize MPER in a lipid context.

## Discussion

The developmental pathways of broadly HIV-1-neutralizing antibodies provide insight into their recombination and maturation and, along with studies of virus-antibody co-evolution, can yield potential strategies for elicitation of similar highly desired antibodies [5,7,38,39]. Prior studies delineating antibody ontogenies have generally employed analysis of B cell immunoglobulin transcripts longitudinally sampled from time of infection antibodies [5,7]. However, such longitudinal data is not available for many broadly neutralizing antibodies. One of these is antibody 10E8, for which samples were collected from only two time points, January and June, 2011. We focused on the January 2011 time point, as it was earlier and was the time point from which 10E8 was isolated through B cell culture. Here we describe a strategy through which we gain insight into the ontogeny of 10E8 from a single time point. Our study highlights the ability to use information from structure, NGS, and function, coupled with tools such as parsimony, bioinformatics sieving, and maximum likelihood, to infer an approximate developmental pathway from a single time point.

The 10E8 antibody is highly mutated (~21% SHM on the heavy chain) and NGS from a cross-sectional time point requires sufficient sampling and a sufficient population of memory B cells expressing low-divergence sequences. In the current study, each heavy chain data set had few low-divergence sequences from the 10E8 lineage. This resulted in inferred UCA and intermediate sequences which were ambiguous, and we resolved this ambiguity through parsimony analysis (S3 and S4 Figs). Additionally, the presence of an unmutated stretch of residues in the CDR H3 region allowed us to identify the HD3-3*01 germline D gene as being substantially conserved from UCA to mature 10E8.

The most parsimonious ontogeny for 10E8 development revealed early mutations in lineage sequences such that by pI1, K52 had changed to a threonine and CDR H3 had incorporated E->K at position 97 and double-proline alterations. Despite these early changes, the functional characteristics of UCA and pI1 were similar (Fig 6A–6C). By pI2 HIV-1 neutralization and tight MPER peptide binding were observed, but not the ability to bind MPER in the liposome context (Fig 6A–6C). Comparison of the CDR H3 sequences of pI2 and pI3 reveal one difference that could underlie this result, a histidine residue at position 98 in pI2 versus a tyrosine at this position in pI3. The charged nature of histidine might reduce recognition in the lipid context, though we cannot exclude the possibility that other sequence differences outside of the CDR H3 loops of the two variants play a role in enabling lipid-context recognition. Overall, MPER peptide affinity correlated with neutralization to a greater degree than binding MPER in the liposome context.

Our findings with the 10E8 lineage have a number of implications for vaccine design. First, UCAs may have substantial clashes with antigen (Fig 2). Lineage analysis suggests that these need to be resolved early in development, and it appears that this can be accomplished by SHM. Second, the 10E8 lineage develops weak affinity for soluble MPER peptide early. Although we were unable to quantify the binding affinity by SPR for UCA and pI1, binding was clearly above background. While weak UCA affinity has been observed previously, the priming antigen for MPER-directed lineages was unclear; here we show that for 10E8 the priming antigen appears to be soluble portions of the MPER. Third, affinity for MPER in the liposome context appeared late at pI3, with pI2 able to neutralize despite poor binding in the liposome context. Thus, either the liposomal MPER binding assay was insensitive to weak liposome-context binding, or liposome binding only occurs later in development. Altogether, the 10E8 lineage information obtained from a single time point thus provides a likely developmental pathway that should inform rational vaccine design efforts.

## Supporting Information

S1 Fig B factor analysis of ligand free 10E8 structures. Crystal structures of the (**A**) 10E8 gHv/gLv (**B**) 10E8 gHv/Mature L and (**C**) 10E8 mature are displayed in B factor putty representation colored from blue to red and with larger and smaller putty size based on atomic B factors scaled from 0 to 40 Å^2^.
(TIF)

S2 Fig Bioinformatics pipeline processing of donor N152 454 Pyrosequencing datasets. (**A**) Read length distribution for published heavy chain NGS data set and the distribution of germline genes associated with reads larger than 300 nt. (**B**) Read length distribution for unpublished heavy chain NGS data set and the distribution of germline genes associated with reads larger than 300 nt. (**C**) Read length distribution for published light chain NGS data set and the distribution of germline genes associated with reads larger than 300 nt. Bars colored in blue denote the germlines of interest.
(TIF)

S3 Fig 10E8 heavy chain ontogeny: lineage members and calculated intermediates. (**A**) ML phylogenetic tree based on VDJ-sieve-selected sequences. (**B**) Sequences belonging to phylogenetically inferred developmental heavy chain intermediates compared to mature 10E8 and constituent germline genes. The coloring scheme for the ML tree was adapted from Zhu et al., [18]. Blue circles indicate nodal points on the tree. Numbers appearing in parenthesis on the left side of the alignment indicate the number of identical residues to the germline V gene.
(TIF)

S4 Fig 10E8 light chain ontogeny: lineage members and calculated intermediates. (**A**) Phylogenetic tree based on CDR L3 signature sieve-selected sequences. To select sequences for ML tree construction, representative sequences were selected randomly from bins of 0.5 divergence units (as shown in left inset). The coloring scheme for the ML tree was adapted from Zhu et al., [18]. Blue circles indicate nodal points on the tree. (**B**) Sequences of phylogeneticaly inferred developmental light chain intermediates compared to mature 10E8 and constituent germline genes. Numbers appearing in parenthesis on the left side of the alignment indicate the number of identical residues to the germline V gene.
(TIF)

S5 Fig Direct binding of 10E8 variants to soluble MPER peptide. Shown are ELISA profiles of antibody variants binding to a biotinylated MPER peptide captured on neutravidin coated plates. Profiles are colored as listed in figure legend. 10E8 UCA and pI1 did not show detectable binding in this format.
(TIF)

S6 Fig Antinuclear autoantigen (ANA) reactivity for UCA and intermediates pI1-pI3. (TIF)

S1 Table IC_50_ values (μg/ml) on a panel of eight viruses for 10E8 revertant and CDR H2 mutants. (DOCX)

S2 Table PCR primers used to prepare samples for 454 pyrosequencing analysis of donor N152. (DOCX)

S3 Table Parameters used to evaluate the fitness of each maturation pathway derived from heavy chain sequences. (DOCX)

S4 Table Parameters used to evaluate the fitness of each maturation pathway derived from light chain sequences. (DOCX)

S5 Table Cardiolipin reactivity for paired heavy and light intermediates. (DOCX)

S6 Table IC50 values (μg/ml) on a panel of eight viruses for pairing of inferred heavy and light chain antibody sequences. (DOCX)
