# Supplementary figures and images for: Developmental Pathway of the MPER-Directed HIV-1-Neutralizing Antibody 10E8
Source: PLoS One. 2016 Jun 14;11(6):e0157409. doi: 10.1371/journal.pone.0157409 (PMC4907498; doi:10.1371/journal.pone.0157409)

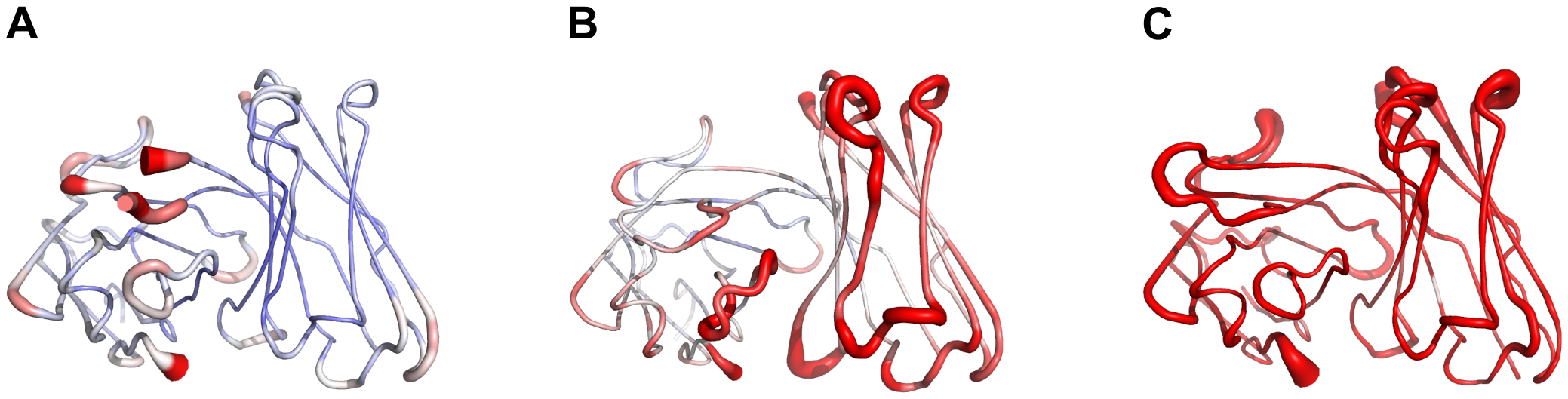

Supplement: S1 Fig — Crystal structures of the (A) 10E8 gHv/gLv (B) 10E8 gHv/Mature L and (C) 10E8 mature are displayed in B factor putty representation colored from blue to red and with larger and smaller putty size based on atomic B factors scaled from 0 to 40 Å2. (TIF) [file pone.0157409.s001.tif]

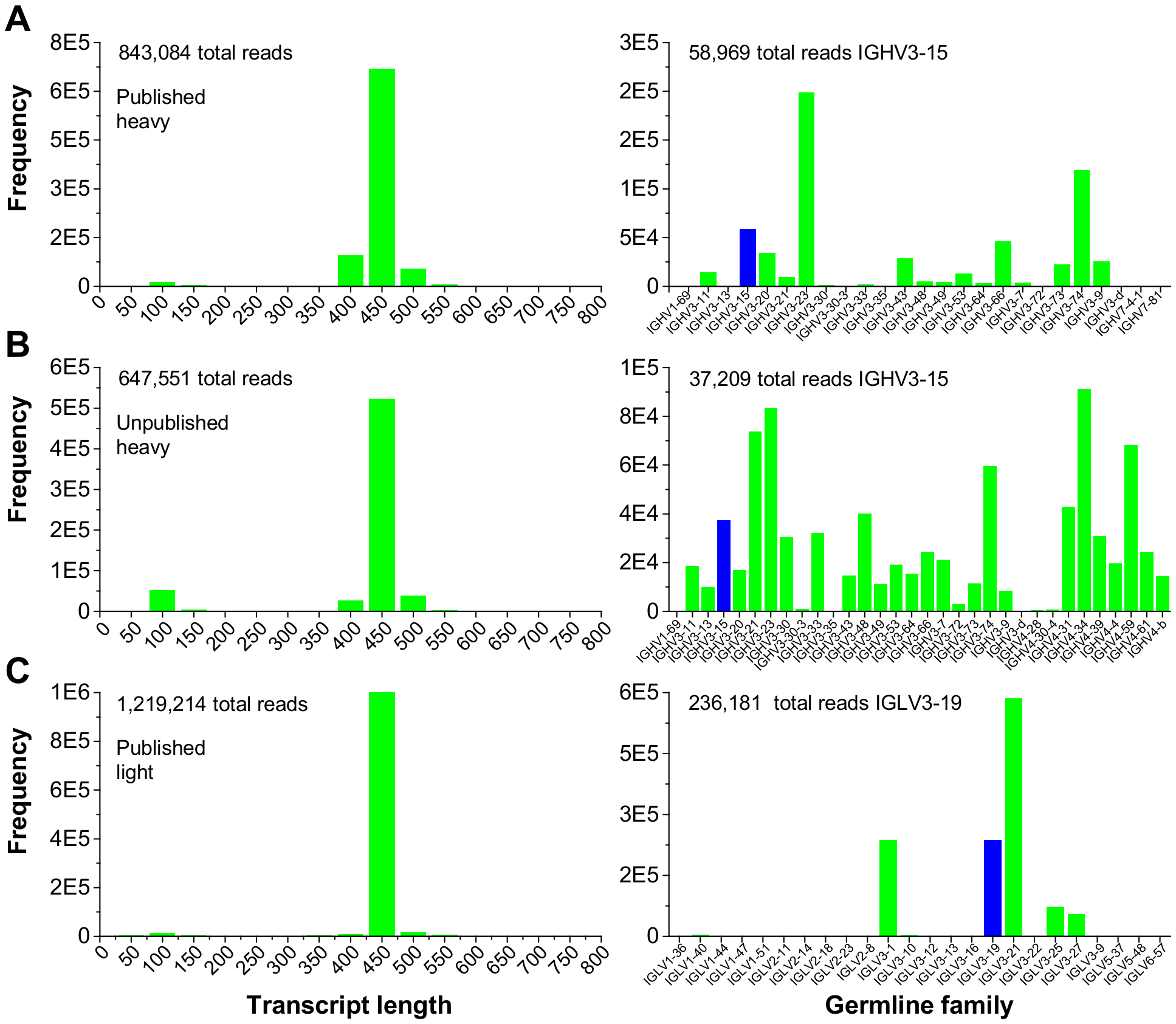

Supplement: S2 Fig — (A) Read length distribution for published heavy chain NGS data set and the distribution of germline genes associated with reads larger than 300 nt. (B) Read length distribution for unpublished heavy chain NGS data set and the distribution of germline genes associated with reads larger than 300 nt. (C) Read length distribution for published light chain NGS data set and the distribution of germline genes associated with reads larger than 300 nt. Bars colored in blue denote the germlines of interest. (TIF) [file pone.0157409.s002.tif]

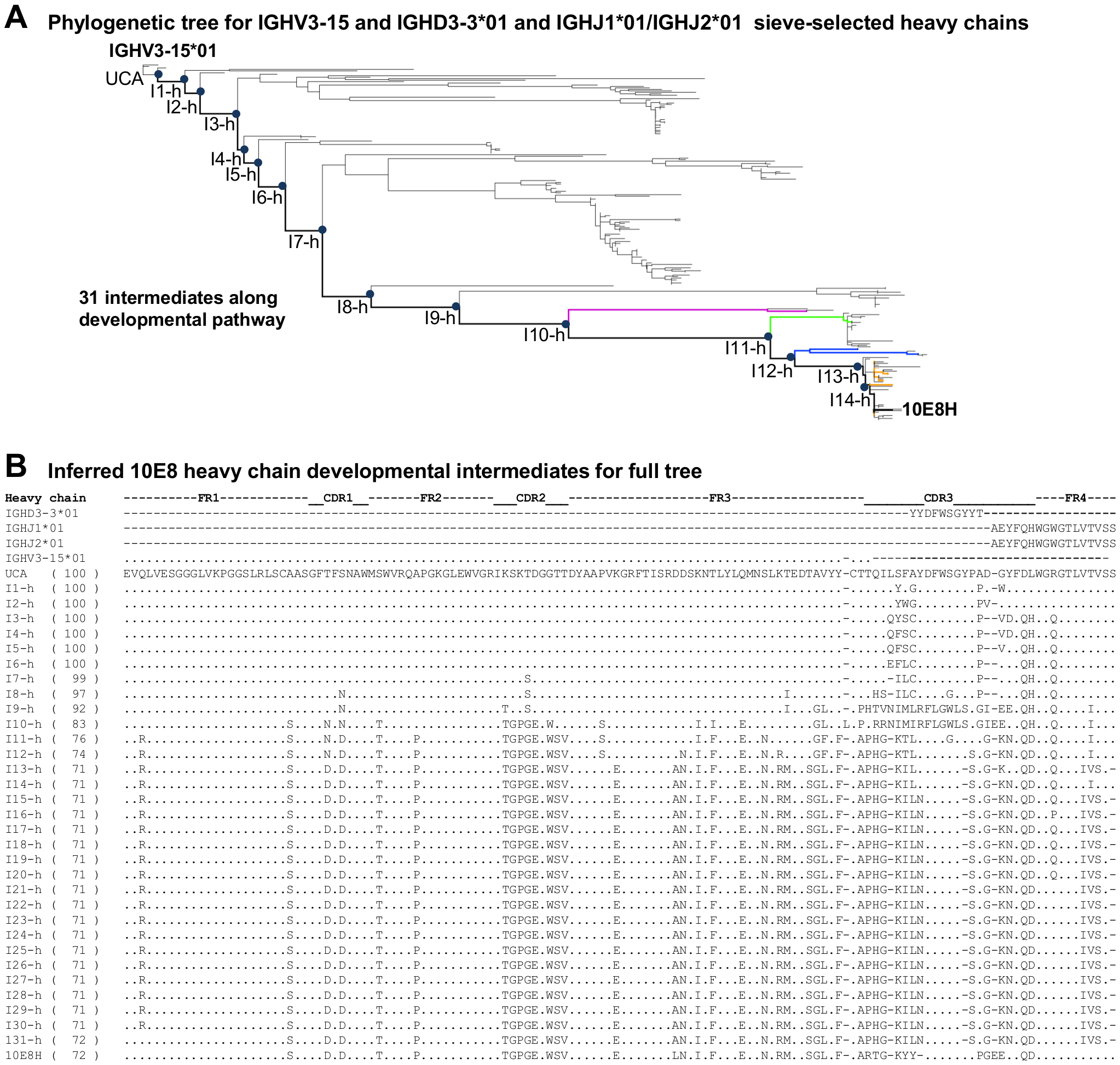

Supplement: S3 Fig — (A) ML phylogenetic tree based on VDJ-sieve-selected sequences. (B) Sequences belonging to phylogenetically inferred developmental heavy chain intermediates compared to mature 10E8 and constituent germline genes. The coloring scheme for the ML tree was adapted from Zhu et al., [18]. Blue circles indicate nodal points on the tree. Numbers appearing in parenthesis on the left side of the alignment indicate the number of identical residues to the germline V gene. (TIF) [file pone.0157409.s003.tif]

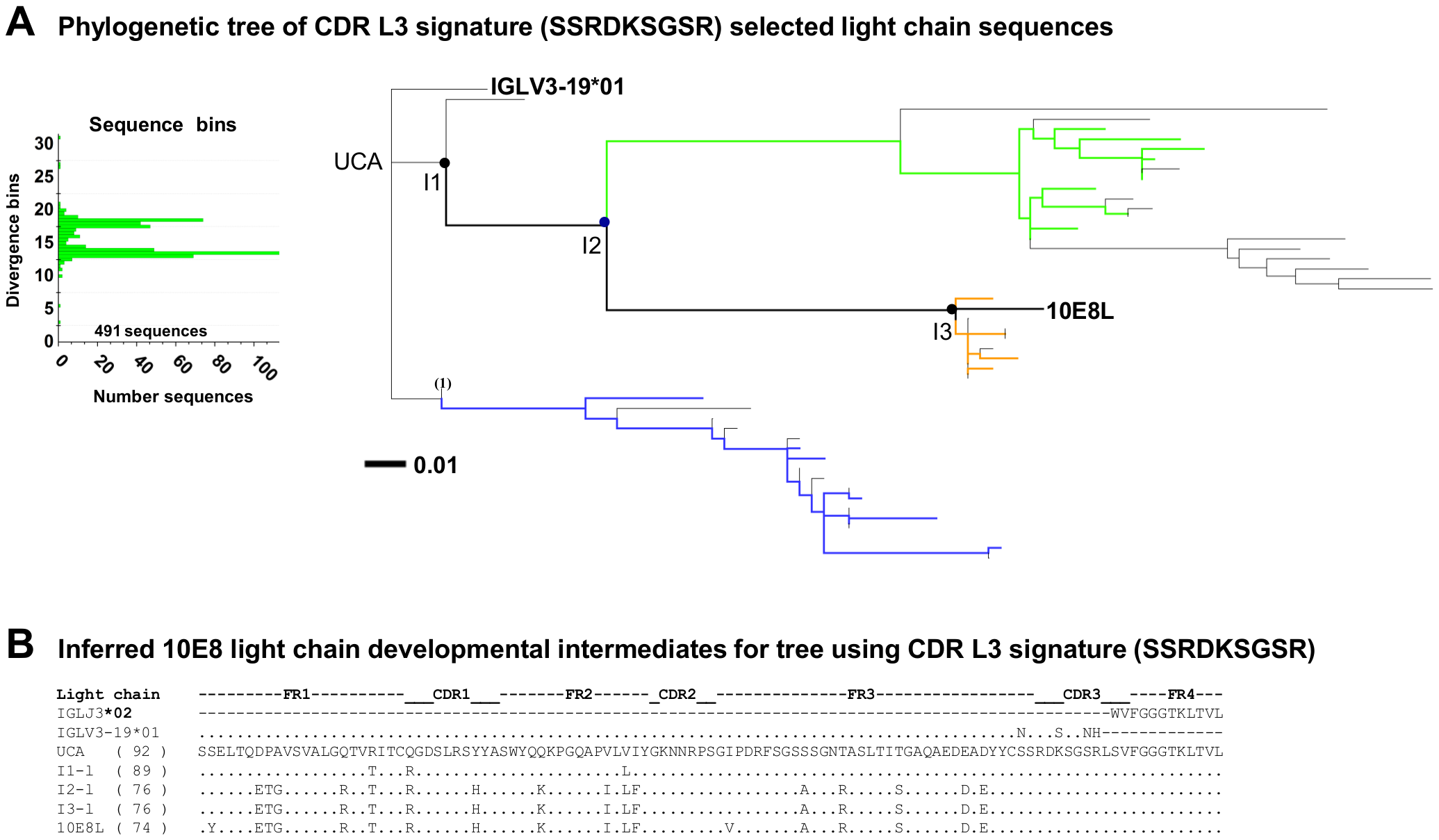

Supplement: S4 Fig — (A) Phylogenetic tree based on CDR L3 signature sieve-selected sequences. To select sequences for ML tree construction, representative sequences were selected randomly from bins of 0.5 divergence units (as shown in left inset). The coloring scheme for the ML tree was adapted from Zhu et al., [18]. Blue circles indicate nodal points on the tree. (B) Sequences of phylogeneticaly inferred developmental light chain intermediates compared to mature 10E8 and constituent germline genes. Numbers appearing in parenthesis on the left side of the alignment indicate the number of identical residues to the germline V gene. (TIF) [file pone.0157409.s004.tif]

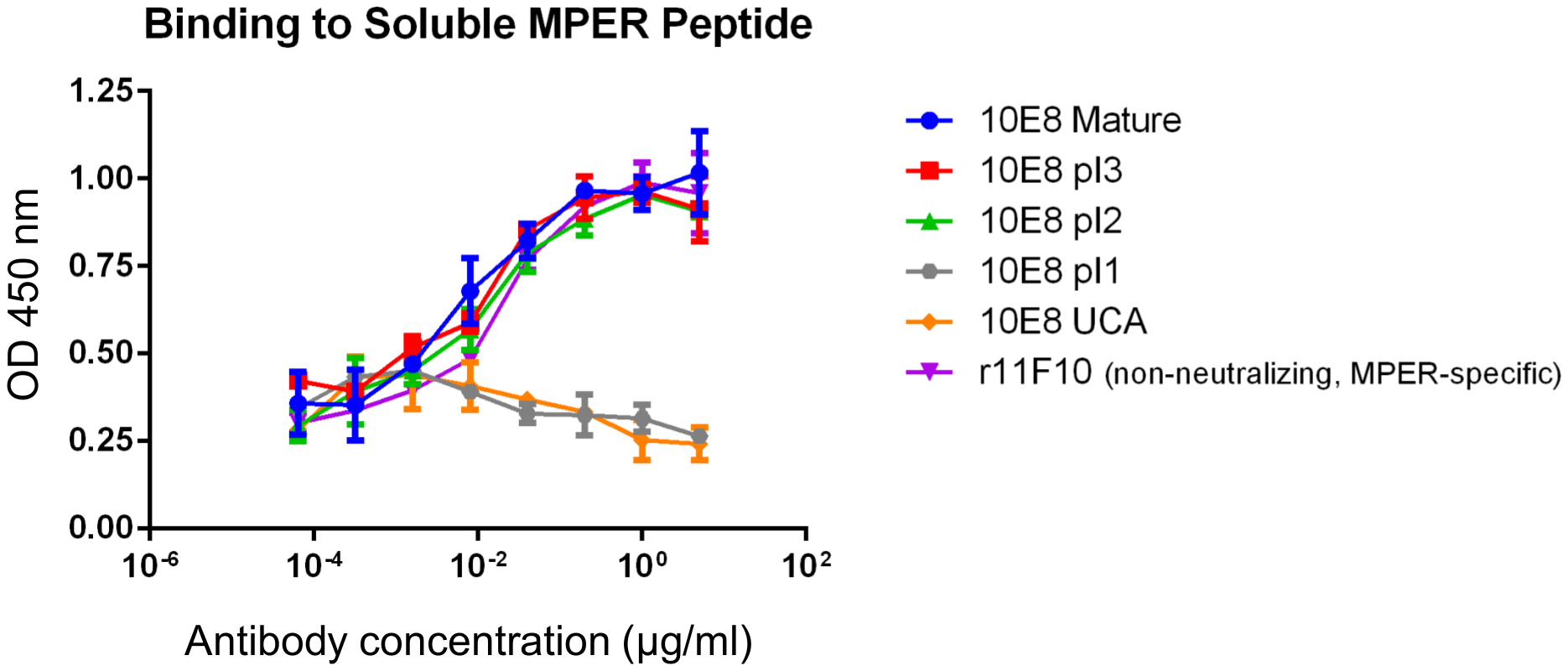

Supplement: S5 Fig — Shown are ELISA profiles of antibody variants binding to a biotinylated MPER peptide captured on neutravidin coated plates. Profiles are colored as listed in figure legend. 10E8 UCA and pI1 did not show detectable binding in this format. (TIF) [file pone.0157409.s005.tif]

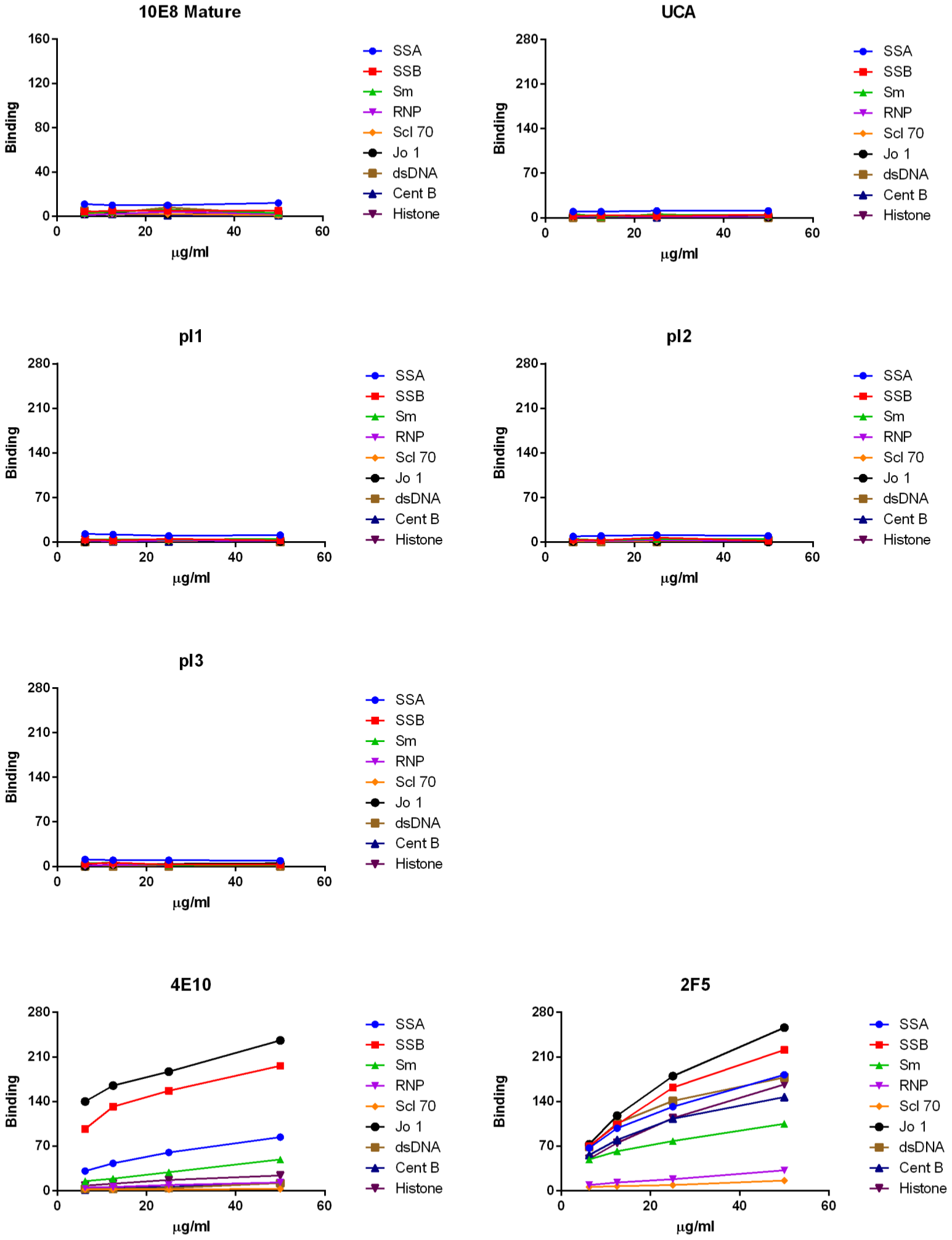

Supplement: S6 Fig — (TIF) [file pone.0157409.s006.tif]
