# Supplementary material for: Developmental Pathway of the MPER-Directed HIV-1-Neutralizing Antibody 10E8
Source: PLoS One. 2016 Jun 14;11(6):e0157409. doi: 10.1371/journal.pone.0157409 (PMC4907498; doi:10.1371/journal.pone.0157409)
Supplement: S1 Table — (DOCX) [file pone.0157409.s007.docx]

**S1 Table. IC_50_ values (μg/ml) on a panel of eight viruses for 10E8 revertant and CDR H2 mutants.**

| **Clade** | **Virus** | **gHv/gLv** | **1H** | **2H** | **4H** | **8H** | **10E8** |
| --- | --- | --- | --- | --- | --- | --- | --- |
| ACD | 6095.V1.C10.SG3 | >50 | 0.066 | 0.024 | >50 | 0.074 | 0.007 |
| AE | CNE59.SG3 | >50 | 1.410 | 0.220 | >50 | 0.078 | 0.008 |
| B | HxB2.DG.SG3 | >50 | 0.635 | 0.131 | >50 | 0.059 | 0.005 |
| B | MN.3.SG3 | >50 | 1.630 | 0.224 | >50 | 0.036 | 0.001 |
| B | 6101.10.SG3 | >50 | >50 | >50 | >50 | >50 | 0.020 |
| BC | CNE40.SG3 | >50 | 0.334 | 0.069 | >50 | 0.159 | 0.007 |
| C | MW965.26.SG3 | >50 | 0.356 | 0.062 | >50 | 0.047 | 0.006 |
| D | NKU3006.ec1.SG3 | >50 | 47.400 | 17.500 | >50 | 9.180 | 1.090 |
