# Supplementary material for: Developmental Pathway of the MPER-Directed HIV-1-Neutralizing Antibody 10E8
Source: PLoS One. 2016 Jun 14;11(6):e0157409. doi: 10.1371/journal.pone.0157409 (PMC4907498; doi:10.1371/journal.pone.0157409)
Supplement: S2 Table — (DOCX) [file pone.0157409.s008.docx]

**S2 Table. PCR primers used to prepare samples for 454 pyrosequencing analysis of donor N152.**

| **Name** | **Primer sequence (5’ 🡪 3’)** |
| --- | --- |
| **Heavy-chain primers** | |
| Forward primer ^a^ |  |
| XLR_A_VH3-15 | CCATCTCATCCCTGCGTGTCTCCGACTCAGGCTATTTTAAAAGGTGTCCAGTGT |
| Reverse primers ^b^ |  |
| XLR-B_3’_CμCH1 | CCTATCCCCTGTGTGCCTTGGCAGTCTCAGGGGAATTCTCACAGGAGACGA |
| XLR-B_3'_γCH1 | CCTATCCCCTGTGTGCCTTGGCAGTCTCAGGGGGAAGACCGATGGGCCCTTGGTGG |

^a^ Forward primer were designed to amplify heavy chain VH3-15 genes.

^b^ μ reverse primers were used in an attempt to capture IgM memory B cells, though only about 100 out of 843,084 total raw heavy chain reads were of IgM origin, none 10E8-like, suggesting that the number of such cells in the periphery is likely low.
