# Supplementary material for: Developmental Pathway of the MPER-Directed HIV-1-Neutralizing Antibody 10E8
Source: PLoS One. 2016 Jun 14;11(6):e0157409. doi: 10.1371/journal.pone.0157409 (PMC4907498; doi:10.1371/journal.pone.0157409)
Supplement: S3 Table — (DOCX) [file pone.0157409.s009.docx]

**S3 Table. Parameters used to evaluate the fitness of each maturation pathway derived from heavy chain sequences.**

| **ML tree** | **Number**  **Intermediates** | **Number**  **Reversions^a^** | **Number**  **N_VD_ additions^b^** | **Number**  **N_DJ_ additions^b^** |
| --- | --- | --- | --- | --- |
| VDJ tree | 31 | 64 | 17 | 10 |
| H3 signature tree | 9 | 10 | 13 | 7 |

^a^ The number of reversions was determined with respect to the UCA and computed using nucleotide sequences.

^b^ N_VD_ and N_DJ_ additions were determined for each UCA using JOINSOLVER (see methods).
