# Supplementary material for: Developmental Pathway of the MPER-Directed HIV-1-Neutralizing Antibody 10E8
Source: PLoS One. 2016 Jun 14;11(6):e0157409. doi: 10.1371/journal.pone.0157409 (PMC4907498; doi:10.1371/journal.pone.0157409)
Supplement: S4 Table — (DOCX) [file pone.0157409.s010.docx]

**S4 Table. Parameters used to evaluate the fitness of each maturation pathway derived from light chain sequences.**

| **ML tree** | **Number**  **Intermediates** | **Number**  **Reversions^a^** | **Number**  **N_VJ_ additions^b^** |
| --- | --- | --- | --- |
| VJ tree | 7 | 2 | 1 |
| L3 signature tree | 4 | 1 | 14 |

^a^ The number of reversions was determined with respect to the UCA and computed using nucleotide sequences.

^b^ N_VJ_ additions were determined for each UCA using JOINSOLVER (see methods).
