# Supplementary material for: Developmental Pathway of the MPER-Directed HIV-1-Neutralizing Antibody 10E8
Source: PLoS One. 2016 Jun 14;11(6):e0157409. doi: 10.1371/journal.pone.0157409 (PMC4907498; doi:10.1371/journal.pone.0157409)
Supplement: S5 Table — (DOCX) [file pone.0157409.s011.docx]

**S5 Table. Cardiolipin reactivity for paired heavy and light intermediates.**

|  | **Antibody Concentration (μg/ml)** | | | | | | | |
| --- | --- | --- | --- | --- | --- | --- | --- | --- |
| **Antibody** | **100.00** | **33.33** | **11.11** | **3.70** | **1.23** | **0.41** | **0.14** | **0.05** |
| 10E8 Mature | 0.0536 | 0.0464 | 0.0373 | 0.0368 | 0.0415 | 0.0390 | 0.0404 | 0.0399 |
| UCA | 0.0388 | 0.0397 | 0.0395 | 0.0369 | 0.0398 | 0.0388 | 0.0378 | 0.0394 |
| pI1 | 0.0418 | 0.0439 | 0.0390 | 0.0398 | 0.0400 | 0.0415 | 0.0377 | 0.0392 |
| pI2 | 0.0578 | 0.0437 | 0.0435 | 0.0433 | 0.0415 | 0.0381 | 0.0391 | 0.0448 |
| pI3 | 0.0530 | 0.0440 | 0.0400 | 0.0392 | 0.0402 | 0.0406 | 0.0398 | 0.0383 |
| 17b | 0.1114 | 0.0580 | 0.0440 |  |  |  |  |  |
| 4E10 | 2.0451 | 2.0326 | 1.9112 |  |  |  |  |  |
| 2F5 | 0.4530 | 0.1444 | 0.0752 |  |  |  |  |  |
