# Supplementary material for: Developmental Pathway of the MPER-Directed HIV-1-Neutralizing Antibody 10E8
Source: PLoS One. 2016 Jun 14;11(6):e0157409. doi: 10.1371/journal.pone.0157409 (PMC4907498; doi:10.1371/journal.pone.0157409)
Supplement: S6 Table — (DOCX) [file pone.0157409.s012.docx]

**S6 Table. IC_50_ values (μg/ml) on a panel of eight viruses for pairing of inferred heavy and light chain antibody sequences.**

| **Clade** | **Virus** | **UCA** | **pI1** | **pI2** | **pI3** | **10E8** |
| --- | --- | --- | --- | --- | --- | --- |
| ACD | 6095.V1.C10.SG3 | >50 | >50 | 0.097 | 0.001 | 0.000 |
| AE | CNE59.SG3 | >50 | >50 | 0.168 | 0.001 | 0.002 |
| B | HxB2.DG.SG3 | >50 | >50 | 0.107 | 0.001 | 0.001 |
| B | MN.3.SG3 | >50 | >50 | 0.008 | 2.0E-4 | 1.0E-4 |
| B | 6101.10.SG3 | >50 | >50 | 1.770 | 0.009 | 0.002 |
| BC | CNE40.SG3 | >50 | >50 | 0.568 | 0.003 | 0.001 |
| C | MW965.26.SG3 | >50 | >50 | 0.482 | 0.001 | 0.001 |
| D | NKU3006.ec1.SG3 | >50 | >50 | >50 | 1.470 | 0.596 |
